# A Case Report: Effect of Robotic Exoskeleton Based Therapy on Neurological and Functional Recovery of a Patient With Chronic Stroke

**DOI:** 10.3389/fneur.2021.680733

**Published:** 2021-07-12

**Authors:** Neha Singh, Megha Saini, Nand Kumar, M. V. Padma Srivastava, S. Senthil Kumaran, Amit Mehndiratta

**Affiliations:** ^1^Centre for Biomedical Engineering, Indian Institute of Technology Delhi (IITD), New Delhi, India; ^2^Department of Psychiatry, All India Institute of Medical Sciences (AIIMS), New Delhi, India; ^3^Department of Neurology, All India Institute of Medical Sciences (AIIMS), New Delhi, India; ^4^Department of Nuclear Medicine and Resonance, All India Institute of Medical Sciences (AIIMS), New Delhi, India; ^5^Department of Biomedical Engineering, All India Institute of Medical Sciences (AIIMS), New Delhi, India

**Keywords:** case report, robotic exoskeleton, stroke, rehabilitation, electromyogram, cortical excitability, fMRI

## Abstract

**Background:** In this study, a novel electromechanical robotic exoskeleton was developed for the rehabilitation of distal joints. The objective was to explore the functional MRI and the neurophysiological changes in cortical-excitability in response to exoskeleton training for a 9-year chronic stroke patient.

**Case-Report:** The study involved a 52-year old female patient with a 9-year chronic stroke of the right hemisphere, who underwent 20 therapy sessions of 45 min each. Cortical-excitability and clinical-scales: Fugl-Mayer (FM), Modified Ashworth Scale (MAS), Brunnstrom-Stage (BS), Barthel-Index (BI), Range of Motion (ROM), were assessed pre-and post-therapy to quantitatively assess the motor recovery.

**Clinical Rehabilitation Impact:** Increase in FM wrist/hand by 6, BI by 10, and decrease in MAS by 1 were reported. Ipsilesional Motor Evoked Potential (MEP) (obtained using Transcranial Magnetic Stimulation) was increased by 98 μV with a decrease in RMT by 6% and contralesional MEP was increased by 43 μV with a decrease in RMT by 4%. Laterality Index of Sensorimotor Cortex (SMC) reduced in precentral- gyrus (from 0.152 to −0.707) and in postcentral-gyrus (from 0.203 to −0.632).

**Conclusion:** The novel exoskeleton-based training showed improved motor outcomes, cortical excitability, and neuronal activation. The research encourages the further investigation of the potential of exoskeleton training.

## Introduction

Post-stroke motor recovery follows a non-linear trajectory (1). Although, there is a period of enhanced plasticity or spontaneous recovery of motor function following a stroke, it is insufficient and often negligible in patients with chronic-stroke. Intensive therapeutic and rehabilitative interventions primarily lead to functional restoration in chronic-stroke survivors (2). While research studies have explored neuronal and motor recovery, patients with chronic-stroke often manifest long-term disability and limitations in the activities of daily living (3). The exact behavior of neurophysiological aspects at a neuronal level showing enhanced responsiveness to treatment in chronic-stroke is not clear yet (1).

Robotic-training for physical therapy is now becoming a new normal for the rehabilitation community (4). It might share a good amount of the clinical load of the therapist and can substantially facilitate the phenomenon of functional neuro-rehabilitation and recovery. An electro-mechanical robotic-exoskeleton was developed for distal joints that synchronize wrist-extension with Metacarpophalangeal (MCP) flexion and wrist-flexion with MCP-extension (4). The exoskeleton targets spasticity through a synergy-based rehabilitation approach while also maintaining patient-initiated therapy through residual muscle activity using Electromyogram (EMG) for maximizing voluntary effort. Here, we present the case of a 52-year old female with late chronic-stroke of 9 years, who had a partial recovery, and its convergent association of potential brain reorganization in response to the novel exoskeleton. The objective of this case study was to explore the neurophysiological repertoire of behavior behind motor recovery in response to the goal-directed treatment using exoskeleton for a patient with chronic-stroke.

## Case Description

The Institutional Review Board (IRB) at the All India Institute of Medical Sciences, New Delhi, India approved the study (protocol number: IEC/NP-99/13.03.2015). The patient provided written informed consent before enrolling in the study.

### Patient

The 52-year old female patient (right-handed) is henceforth referred to as Mrs. X. She was well-educated and an airline pilot by profession. She has a family history of stroke; her mother suffered from a stroke. There was no other relevant genetic or psychosocial history. She survived acute right Anterior Cerebral Artery (ACA) and Middle Cerebral Artery (MCA) ischemic infarct and a small left ACA infarct in April 2009. She was conscious at the time of stroke onset. She was immediately admitted to ICU in a local hospital in Delhi. She was conscious, oriented, and cooperative. Her MRA, MRV, and neck angio, ECG, ECHO, and Holter monitoring were normal. She had gaze preference to right. After 2.5 h of admission, she developed jerky movements in the left upper-limb and was loaded with injection epsolin 1,000 mg. The infarct resulted in left hemiparesis, less control, and functional outcomes in the left-limb with power-0/5. The power in the right-limb was 5/5. She also presented left facial palsy, weak left eye closure, and slurring of speech. She did not have health issues like hypertension, diabetes, tobacco smoking, or alcohol but had a history of diabetes in the family (mother).

**Figure 1:**
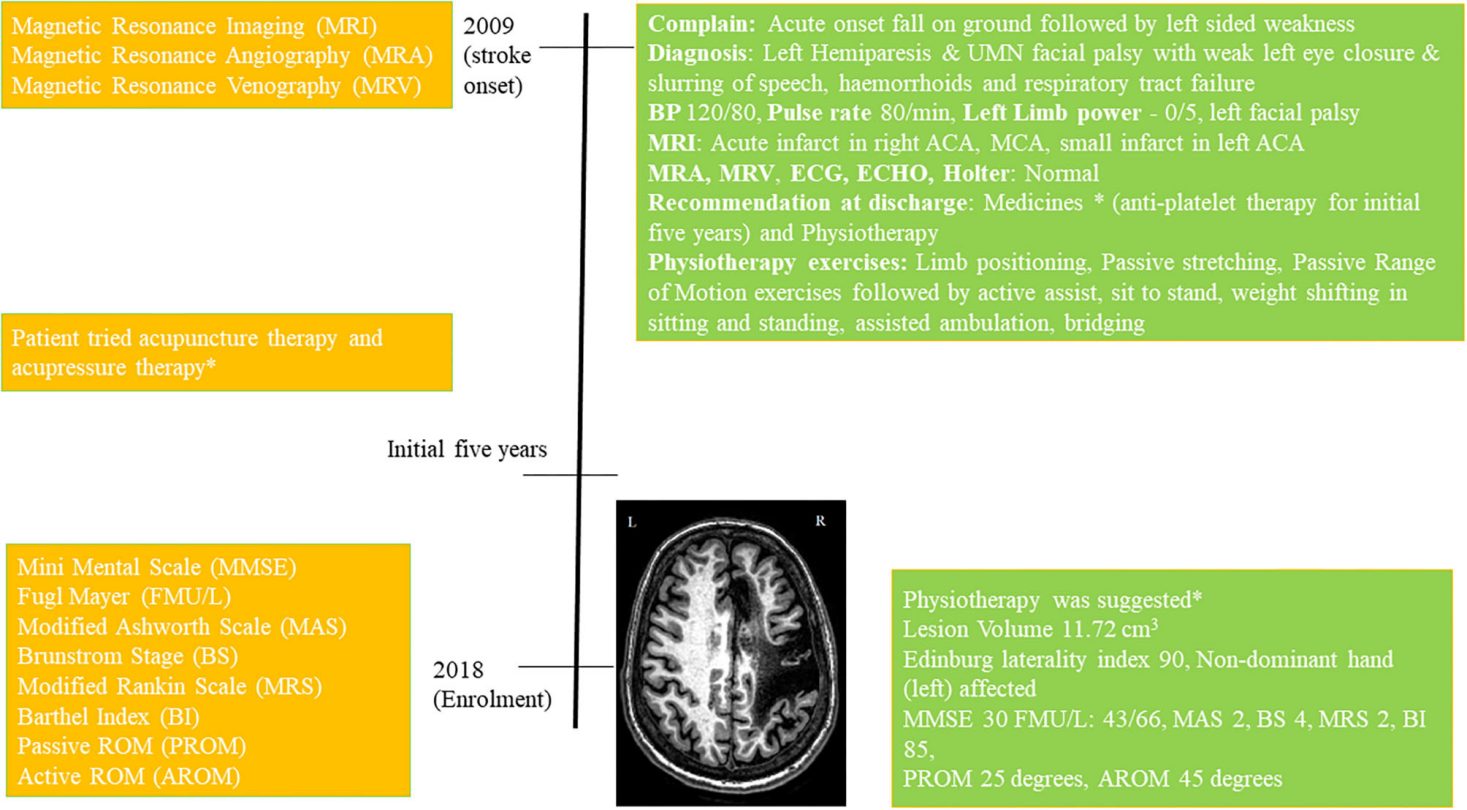
Timeline with relevant data from the episode of care, *details in Supplementary Material.

She was moved to the ward after 4 days and discharged to home in stable condition after 15 days. She was on antiplatelet therapy for 5 years and later she continued prophylactic antiplatelet therapy (details in Supplementary Material). She underwent physiotherapy immediately following her discharge and had acupressure-therapy, acupuncture-therapy, home-based exercises for an initial few (~5) years before enrolling in this study, (Timeline Figure 1, full details in Supplementary Material).

At the enrolment in 2018, the volume of the lesion was 11.72 cm^3^, Edinburg laterality-index was 90 (non-dominant hand affected) and Mini-Mental Score Examination (MMSE) was 30. She was given physiotherapy exercises for home (details in Supplementary Material). The patient scored Modified Ashworth-Scale (MAS) 2 at wrist-joint, Brunnstrom Stages (BS) 4, Modified Rankin Scale (MRS) 2, Barthel Index (BI) 85, upper-limb Fugl Meyer (FM) scale 43/66, lower-limb FM scale 29/34, at wrist joint Passive ROM (PROM) 45°, and Active ROM (AROM) 25°.

### Therapy Protocol

#### Robotic-Therapy-Sessions

The device (Supplementary Figure 1) is actively initiated by EMG activity of Extensor Digitorum Communis (EDC) muscle with robot motion triggered only if the EMG thresholds are crossed and it provides an interactive adaptive performance visual biofeedback in real-time. At baseline position, the patient tries to extend the wrist voluntarily for the first 3 s after the green LED cue. If the EMG crosses the predefined threshold, the exoskeleton will be triggered for an assisted wrist extension and finger flexion movement. Once it reaches the final position, the exoskeleton then assists the patient's hand back to the baseline position, wrist flexion with finger extension. Simultaneous with this motion assistance, the performance feedback is given to the patient in real-time. The device was patient-specific as has flexibility in accommodating patients as per the varying clinical presentation, with customizable motion-parameters: (i) initial-position for a range of motion (ROM), (ii) final-position for ROM, (iii) speed, (iv) residual muscle-activity, and (v) height of finger-support (4). It is a simple and easy-to-operate exoskeleton with a user friendly interface of LCD and knobs for inputs, as presented in the device paper by Singh et al. (4). The configurability of the threshold was adjusted during the study manually and individually using the BIOPAC MP150 EMG acquisition software according to the residual EMG activity of an individual patient with the advantage of making the system patient-specific by including patients with minimal residual muscle-activity in the protocol. Pre to post-therapy, the amplitude of the EMG threshold changed from 0.4 to 0.6 V (amplified with gain = 2,000, Band Pass Filter = 10–500 Hz, Notch Filter = 50 Hz, Sampling Frequency = 1,000 Hz) (4).

Robotic training was given for 45-min a day for 20 sessions, with ~250 trials of 10 s each undertaken (4). The therapy sessions were given by the trained physiotherapists with more than 5 years of experience in stroke rehabilitation. Mrs. X completed this therapy in clinical settings in 31-days with pre-to-post-therapy clinical data acquired on day-1 and day-32, respectively. She adhered well to the therapy and tolerated therapy with no adverse effects. There was no change in the therapy session for the whole 20 sessions. As the device is easy to operate, she took interest in operating it on her own. The patient's peripheral vision was very strong, probably because of her occupation (airline pilot by profession) and she was able to see visual feedback even when she was not actively watching it. Though, she had no contractures, she chose 45° as range-of-motion (ROM) on day-1 with speed being constant (30°/s), as she felt comfortable. Due to flexor-hypertonia, active-ROM (AROM) of the wrist was 30°, started from −5° flexion to 25° extension. While wearing the robotic exoskeleton in baseline position with wrist and fingers tied up with Velcro-straps, her wrist was maintained at −5° flexion. The time required by the patient to put the exoskeleton on was 105-s and taking off the exoskeleton took 22-s on day 1.

#### Pre and Post-therapy Clinical-Data Acquisition

Clinical scales including MAS, BS, MRS, BI, FM scale, Passive ROM, and Active ROM, were acquired at day 1 and day 32. On day-32, a self-designed subjective feedback-form, and System Usability Scale (SUS), and question-answer session were also undertaken to gain the patient's perspective of the device (Supplementary Tables 1, 2 and Subjective Questions). SUS form, a standard reliable method for measuring product usability of a novel developed product across a wide range of industries (5), was also obtained from the patient. The SUS scores ranged from 80 to 100, a score of 87.5 from the patient, reflecting the promising scope of “acceptance” and usability from the patient score, which ranged from 80 to 100 (Supplementary Table 2).

Mrs. X was compliable with Transcranial Magnetic Stimulation (TMS). Single-pulse TMS stimuli were applied at 100% Motor Threshold with the procedure widely used (6), using a flat 70 mm figure-of-eight coil [type-D70 (AC), Magstim Rapid^2^, UK] from EDC muscle (Details in Supplementary Material). Five MEP signals out of 10 consecutive trials were averaged. Cortical-excitability measures, Resting Motor-Threshold (RMT), and Motor Evoked Potential (MEP) on cortical representation area of EDC muscle on the ipsilesional and contralesional-hemisphere, were obtained as per the procedure described in the literature including our previous study (6).

Structural T1 image (Supplementary Figure 2) and fMRI BOLD images were acquired for affected and unaffected hand movement, using 3T MR-scanner (Achieva 3T TX, M/s. Philips Healthcare). Patient repeated self-paced sequential-maximum extension and flexion of the wrist in block-design paradigm using the affected and unaffected hand (separately). Data-analysis (in SPM12) included realignment by aligning images to mean-image, co-registration using T1-image, normalized and smoothing with 8 × 8 × 8 Full Width at Half-Maximum (FWHM) filter on pre-and post-BOLD acquisitions. The Talairach client was used to correlate the MNI coordinates for further analysis.

### Clinical Rehabilitation Impact

The protocol was smooth and Mrs. X tolerated the therapy sessions well and had no complaints. An SUS score value of 87.5, subjective feedback, and questions-answers were obtained after 20 sessions (Supplementary Tables 1, 2).

#### Clinical Scores

Clinical scales and cortical-excitability measures for the patient pre-and post-therapy are outlined in Table 1. The reduction in impairment was quantitatively observed to be increased in upper-limb FM scale value by 9 units, an increase in the distal-component of FMW/H was 6 units. Spasticity decreased from the MAS value from 3 to 2 (Table 1). BI increased from a value of 85 to 95 with no change in BS and Mrs. X PROM was observed to improve by 15° at wrist joint. AROM was observed to increase by 5° at wrist joint. Pre-therapy, she used to start wrist-flexion from −5°, however, in post-therapy-sessions she was able to start the wrist-extension from 0° only, because of release in flexor hypertonia. After the 20th therapy session, she had set the ROM of the robotic-exoskeleton at 60° (day-1 ROM 45°) at the same speed.

**Table 1:** Details of clinical scales, cortical excitability measures.

**Clinical scales**	**Pre**	**Post**	**Difference**	**Cortical excitability**	**Pre**	**Post**	**Difference**
FM	43	52	9 (↑)	Ipsilateral MEP (μV)	108	206	98 (↑)
FM (W/H)	9	15	6 (↑)				
F M (S/E)	34	37	3 (↑)	Ipsilateral RMT (%)	85	79	6 (↓)
MAS	2	1	1 (↓)				
BS	4	4	0	Contralateral MEP (μV)	84	127	43 (↑)
BI	85	95	10 (↑)				
MRS	2	2	0	Contralateral RMT (%)	80	76	4 (↓)
AROM	^*^25°	^**^30°	5° (↑)				
PROM	45°	60°	15° (↑)	Normalization (RMT ratio)	1.062	1.039	0.023 (↓)

*FMA (max 66): Fugl-Meyer Assessment, FM W/H (max 24): Wrist / Hand component of FMA, S/E (max 42): Shoulder/Elbow component of FMA, MAS (0–4): Modified Ashworth Score, BS (max 7): Brunnstrom Stages, BI (max 100): Barthel Index, MRS (max 5): Modified Rankin Scale, Passive ROM (max 70): Active ROM (max 70), MEP: Motor Evoked Potential (in μV), RMT: Resting Motor threshold (in %, max 100), Normalization (RMT ratio) = Ipsilateral RMT/Contralateral RMT*.

* *Starts from −5° wrist flexion*.

** *Starts from 0°, (↑) indicates increase, (↓) indicates decrease*.

#### Cortical-Excitability Measures

Pre- to post-therapy, the ipsilesional RMT was decreased by 6%, and MEP amplitude was increased by 98 μV with muscle contraction response being observed in the dorsal wrist and third digit in more than 5 out of 10 consecutive attempts. Pre to post-therapy, the contralesional RMT decreased by 4%, and MEP amplitude increased by 43 μV with muscle contraction response observed in the dorsal wrist (Table 1). The relative % change, expressed in terms of percentages as the ratio of the difference between post-therapy and pre-therapy scales normalized to their pre-therapy scales, pre to post-therapy, for MEP amplitude showed a 90.7% increase for ipsilateral-hemisphere and 51.1% increase for the contralateral-hemisphere, and for RMT was 7% decrease for ipsilateral-hemisphere and 5% decrease for the contralateral-hemisphere. RMT-asymmetry, a ratio of ipsilateral RMT and contralateral RMT, indicated a trend towards normality (close to 1) decreasing from 1.062 to 1.039 post-therapy (Table 1).

#### fMRI Measures

**Figure 2:**
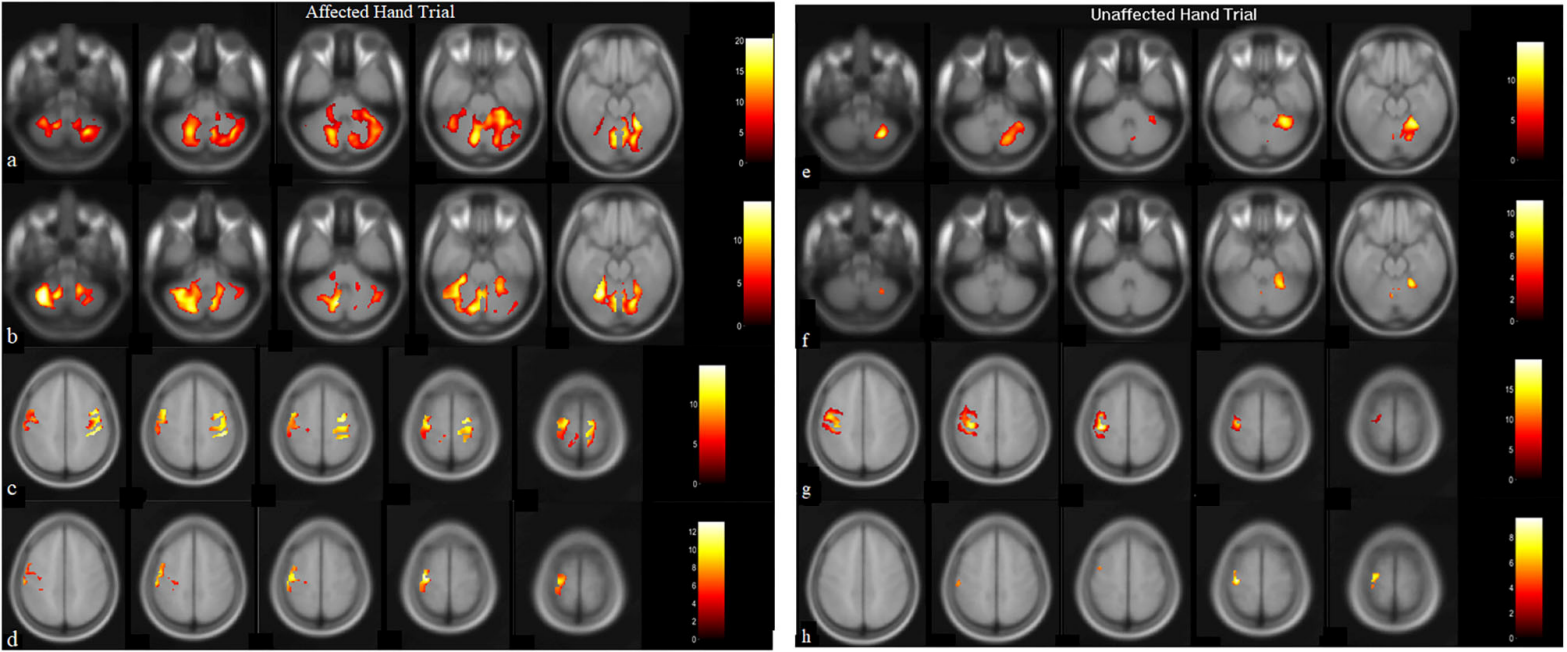
BOLD images for wrist extension task with voxel level threshold *p* < 0.05 (FWE-corrected) and cluster size threshold of 10 voxels. Thirty-one ascending transversal slices, repetition time (TR): 2,000 ms, field of view (FOV): 230 × 230 × 155 mm, Flip-angle: 90 degrees, voxel size: 1.8 × 1.8 × 5 mm and echo time (TE) = 30 ms. Presentation on MR compatible 20” LCD monitor (Esys *in vivo* eprime 1.1) with projection on mirror attached to head coil where image-of-hand notifies the active-block and cross on image-of-hand represents the rest-block. Data-analysis (in SPM12) included realignment by aligning images to mean-image, co-registration using T1-image, normalized, and smoothing with 8 × 8 × 8 Full Width at Half-Maximum (FWHM) filter on pre and post-BOLD images. Talairach-client was used to correlate MNI-coordinates with gray and white matter. **(a,c)** are pre-robotic training images, **(b,d)** are post-robotic training images during affected-hand trial, **(e,g)** are pre-robotic training images and, **(f,h)** are post-robotic training images of masked regions cerebellum (−52:8: −20 slices) and sensorimotorcortex (48:6:72 slices) during the unaffected-hand trial (*p* < 0.05).

Post-therapy, during the affected-hand trial, the number of activated voxels were observed to substantially decrease in ipsilesional sensorimotor-cortex (SMC) - precentral (from 1346 to 114) and postcentral-gyrus (from 914 to 100) (Figure 2, Table 2). Reduction in contralesional precentral (from 991 to 665) and postcentral-gyrus (from 605 to 444) were also observed. Though, there was a decrease in ipsilateral activated voxels, a considerable reduction in Laterality-index (LI), ranged from 1.0 (all contralateral activation) to −1.0 (all ipsilateral activation), of sensorimotor-cortex (SMC) was also observed in precentral (from 0.152 to −0.707) and postcentral-gyrus (from 0.203 to −0.632). This decrease in laterality index demonstrates a substantial decrease in the number of contralateral activated voxels. A large activation increase in ipsilateral-cerebellum (CBM) exterior (from 1908 to 3395) and a decrease in contralateral-CBM exterior (from 4502 to 2261) was observed post-therapy. An activation decrease in contralateral CBM white-matter (from 999 to 462) was also observed. Ipsilateral cerebellum-ratio was observed to substantially increase in CBM exterior (from 0.298 to 0.6) and CBM white-matter (0.528 to 0.69), indicating an increased ipsilateral CBM activation.

**Table 2:** Pre and post-robotic sessions Blood Oxygen Level Dependent (BOLD) activation pattern in affected and unaffected hand trials in mask regions.

**Mask regions**	**Pre-therapy**					**Post-therapy**				
	**No. of voxels**	**Threshold**	**No. of voxels**	**Threshold**	**LI/Ips CB ratio**	**No. of voxels**	**Threshold**	**No. of voxels**	**Threshold**	**LI/Ips CB ratio**
**Task by the affected hand**										
Hemisphere	Right		Left			Right		Left		
Precentral gyrus	1,346	14	991	14.8	0.152	114	9.4	665	13.1	−0.707
Postcentral gyrus	914	14.2	605	9.5	0.203	100	9.7	444	10.9	−0.632
CBM exterior	4,502	20.3	1,908	17.5	0.298	2,261	12.3	3,395	14.5	0.6
CBM white matter	999	15.2	1,116	17.1	0.528	462	10.2	1,030	13.6	0.69
**Task by the unaffected hand**										
Precentral gyrus	247	8.6	930	19.9	0.580	0	0	168	9.5	1
Postcentral gyrus	13	5.5	655	17.4	0.961	0	0	58	6.2	1
CBM exterior	1,760	14.7	27	7.6	0.98	341	11.1	19	6.7	0.94
CBM white matter	320	9.7	0	0	1	83	7.7	8	5.2	0.91

*CBM, Cerebellum; Laterality Index (LI) = (contralateral voxels – Ipsilateral voxels)/(contralateral voxels + Ipsilateral voxels); Ipsilateral cerebellum (Ips CBM) ratio = Ipsilateral cerebellum voxels/(Ipsilateral cerebellum voxels + Contralateral cerebellum voxels)*.

Post-therapy, during the unaffected hand trial, the number of activated voxels were decreased in contralateral/contralesional SMC - precentral (from 930 to 168) and postcentral-gyrus (from 655 to 58) (Figure 2, Table 2). Reduction in ipsilateral/ipsilesional precentral (from 247 to 0) and postcentral-gyrus (from 13 to 0) were also observed. With ipsilateral voxels reduced to zero, the LI of the SMC was observed to increase in precentral (from 0.58 to 1) and postcentral-gyrus (from 0.961 to 1). Ipsilateral cerebellum-ratio was observed to decrease in the CBM exterior (from 0.98 to 0.94) and CBM white-matter (from 1 to 0.91).

## Discussion

### Changes in Clinical Scores

Mrs. X demonstrated a substantial reduction in impairment as seen with the improvement in clinical scales: increase in FMU/L by 9, BI by 10, and decrease in MAS by 1. Out of a total 9 units increase in FMU/L, the Wrist/Hand component of FM increased by 6 units indicating an enhanced distal functionality post-therapy. As shown in the studies by Gladstone et al. (7) and Shin et al. (8), a value of 6.6 on a scale of 66 (FMU/L) reflects the potential Minimally Clinically Important Difference (MCID) and in this study, FMU/L and FMW/H reached the MCID. An increase in passive and active ROM gave increased the degree of movement in the wrist to confidently participate in ADL, as evidenced by an increase in BI by 10 units. She was able to do activities such as opening and closing clips (for drying clothes), held the plates straight while carrying them, etc., which she was not able to do pre-therapy.

### Changes in Cortical-Excitability

An increase in cortical-excitability in both hemispheres might suggest an increase in neuroplasticity and motor-cortex excitability in terms of a decrease in RMT and an increase in MEP amplitude for EDC muscle cortical-representation (hotspot), a muscle involved in exoskeleton-training (9). The changes in the threshold were most likely due to the intervention received rather than inter-session variability as MEPs, which were acquired at two time points with an interval of 31 days: day 1 (pre-therapy) and day 32 (post-therapy) (10–13). Moreover, critical studies like that by Hendrics et al., and Jong et al., have established MEP as a sensitive and valid prognostic marker of motor recovery after stroke (14–16). For cortical-excitability to be increased in the ipsilesional-hemisphere for patients with stroke (after recovery), the ipsilesional-RMT should be decreased from pre-to-post-therapy and hence, normalization or RMT ratio (RMT Ipsilesional/RMT contralesional) should decrease to approach normalization (17–20). A reduction in interhemispheric-asymmetry was observed in the normalization ratio, from pre-to-post-therapy (Table 1), from 1.062 to 1.039 with a mean decrease of 0.023, indicated toward a trend of normalization. A potential increase in cortical-excitability in the ipsilesional-hemisphere might suggest restoration and improvement in the functional integrity of corticospinal tract as functional recovery potential in chronic-stroke depends largely on the integrity of these tract (21). The recruitment of perilesional areas or exploitation of preserved functional recovery reservoir in the ipsilesional-hemisphere may be attributed to the normalization of the RMT ratio (17, 22).

### Changes in fMRI Activations

Increased cortical excitability was paralleled by the observed reduced BOLD signal intensity in both hemispheres. With intact MEP at the ipsilesional-hemisphere pre-therapy, ipsilesional-SMC displaying substantially reduced activation after the intervention; putatively reflects improved synaptic efficiency (23). Reduction in motor-cortex activations post-therapy might correspond to strengthened synaptic efficiency modulated by repetitive task-oriented exoskeleton training. The laterality-index of SMC was also observed to decrease in precentral (from 0.152 to −0.707) and postcentral gyrus (from 0.203 to −0.632) which might be demonstrating a substantial decrease in the number of contralateral activated voxels and shifting of cortical-reorganization from contra to ipsilateral-hemisphere. A considerable reduction in LI of SMC (change in LI: ΔLI postcentral = 0.835 and ΔLI precentral = 0.859), especially with decreased contralateral-hemisphere activations indicates that the patient achieved skilled motor performance (24). To the best of our knowledge, a change of ~0.85 in LI has never been reported in the literature with any intervention or conventional therapy, however, any direct comparison would be inappropriate, considering different factors like chronicity, site of lesion, age, etc. It is plausible that exoskeleton training might have promoted use-dependent reorganization during motor training, resulting in shifting of activation from contra to ipsilateral corresponding to good recovery (vicariation) (18). It might indicate that with focused exoskeleton training the potential recovery might be accelerated. Few studies in the wider literature have explored LI through fMRI activation for affected-hand pre and post rehabilitation intervention, except Brain Computer Interface (reported ΔLI~0.23), Constrain Induced movement therapy (ΔLI~0.25), and low-frequency repetitive TMS over the contralateral hemisphere of the primary motor area (ΔLI~0.14) (25– 27).

Prominently increased cerebellar-motor activation in the Left-CBM exterior ipsilateral to the affected hand could be associated with the recovery process reinforcing CBM's postulated role in motor learning (28). The increased ipsilateral cerebellum-ratio during the affected-hand trial could potentially be a possible consequence of increased cerebellar-cerebral functional connectivity (28). SUS score (of ~87.5), falling in the “excellent-acceptability” category, along with subjective feedback and question answers exhibited acceptance of the robotic-exoskeleton in clinical settings (Supplementary Tables 1, 2, Subjective Questions asked).

Compared with other available wrist rehabilitation devices, the HWARD robot saw an improvement in FMW/H (~4) post-therapy. The SMC laterality index represents a shift in interhemispheric balance over time from the contralateral to the ipsilateral side (29). The Hand Mentor Pro robot observed improvement with FMW/H being 5.6, FMU/L 10.33 in combination with a home exercise program (which alone reported FMW/H 4.9 and FMU/L 9.3) in 99 patients with stroke (30). The reported gain post Constraint-Induced Movement therapy (CIMT), was FMU/L ~13 and BI ~13.5 (31). Furthermore, a meta-analysis and systematic review of CIMT evidenced an enhancement in FMU/L and Action Research Arm Test (ARAT) scores with improved hand control, arm placement, and increased strength as compared to standard therapy in patients with subacute and chronic-stroke (32). With sparse literature exploring cortical-excitability changes in the lower-limb (33) and upper-limb (34), virtual mirror task with biofeedback observed an increased MEP by up to 46.3% (95% CI: 30.4~80.0) compared with the real mirror task (34). The observed increase in FMW/H of patients with chronic-stroke in robotic-assisted wrist-training and dose-matched conventional intervention was ≤ 4 (8, 35–38), however, any direct comparison would be inappropriate considering different factors like chronicity, site of lesion, and age, etc.

Although there are research studies targeting improvement in motor functions in chronic stroke survivors, most of the patients are left with long-term disabilities (3). Our data imply that 4 weeks of focused motor-learning training (with only 20 sessions of 45 min each) using novel voluntary muscle-activity triggered goal-directed exoskeleton is capable of producing clinically relevant neuroplasticity in terms of cortical-excitability and LI change (of ~0.85 in SMC) in functional MRI activation even in chronic-stroke as long as 9 years when any improvement in motor performance is likely to be attributed to being exercise-induced rather than spontaneous recovery. Various strategies could have enhanced the clinically relevant neuroplasticity e.g., target movement of robotic-exoskeleton was specific, measurable, achievable, repetitive, and timed (39), reinforced with maximizing voluntary residual muscle-activity combined with real-time visual performance-biofeedback and proprioceptive-feedback for sensorimotor-integration in every cycle of the movement as was also reported by (40, 41). However, the exoskeleton is in the prototype stage, and the Biopac EMG system was used in the data acquisition for research and validation. In the future, the EMG system should be replaced by a lightweight EMG amplifier to make the whole system compact. The device needs to be further optimized in terms of weight and aesthetics in order to be used for home-based rehabilitation in the future. This case study provided distinct dynamics of post-stroke recovery that deserve further investigation using a larger sample and examining the potential of the exoskeleton.

### Limitations

The study lacks mid-term clinical assessment, and long-term follow-up of the patient, and activity level measurements like Wolf-Motor Function test and Action-Research Arm Test, Functional Independence Measure, Motor Activity Log, and Stroke Impact Scale, etc.,

## Conclusion

The potential of the robotic exoskeleton must be considered further for accelerating post-stroke motor recovery.

## Data Availability Statement

The raw data supporting the conclusions of this article will be made available by the authors, without undue reservation.

## Ethics Statement

The studies involving human participants were reviewed and approved by Institute Review Board, All India Institute of Medical Sciences, New Delhi, India. The patients/participants provided their written informed consent to participate in this study. Written informed consent was obtained from the individual(s) for the publication of any potentially identifiable images or data included in this article.

## Author Contributions

NS and AM conceptualized and designed the study. AM led the study, provided the scientific inputs, and reviewed the multiple iterations of the manuscript with NS. NS performed a literature survey, developed the device, data analysis, data interpretation, and wrote the manuscript. MS performed patient recruitment, robotic therapy, and data collection. NK, SK, and MVPS provided the scientific inputs, clinical support, and clinical resources for experiments. All authors read and approved the final version of the manuscript.

## Conflict of Interest

The authors declare that the research was conducted in the absence of any commercial or financial relationships that could be construed as a potential conflict of interest.
