# Supplementary material for: A Case Report: Effect of Robotic Exoskeleton Based Therapy on Neurological and Functional Recovery of a Patient With Chronic Stroke
Source: Front Neurol. 2021 Jul 12;12:680733. doi: 10.3389/fneur.2021.680733 (PMC8313089; doi:10.3389/fneur.2021.680733)
Supplement: Supplementary file 1 [file Table_1.docx]

**Supplementary Material**

**Patient Details:**

*Age*: 52 (at the time of Enrolment)

*Sex*: Female

*Handedness*: Right

*Occupation*: Airline pilot by profession

*Family history of stroke:* Her mother

*Relevant genetic or psychosocial history*: Not available

**Details at the Onset of Stroke (April 2009):**

*Complain*: Acute onset fall on ground followed by left sided weakness

*Diagnosis*: Left Hemiparesis & UMN facial palsy with weak left eye closure & slurring

of speech, haemorrhoids and respiratory tract failure

*BP:* 120/80, *Pulse rate*: 80/min, *Left Limb power* - 0/5, Left facial palsy with weak eye closure

*MRI*: Acute infarct in right ACA, MCA, small infarct in left ACA

*MRA, MRV, ECG, ECHO, Holter:* Normal

*State*: Consciousness, cooperative oriented

*History of hypertension, diabetes, tobacco smoking or alcohol*: No

*Prior Transient Ischemic attack (TIA):* No

Infarct resulted in left hemiparesis, less control, and functional outcomes in the left-limb with power-0/5.

*Recommendation at discharge:* Medicines (anti-platelet therapy for initial five years) and Physiotherapy

*Details of Medication from onset to completion of robotic therapy:*

1. Prevagold (Aspirin, Clopidogrel and Rosuvastatin)
2. C BioD3
3. Calcirol

Note: Aspirin and Clopidogrel are dual antiplatelet therapy agents that help to prevent blood clot, Rosuvastatin belongs to statin group for lipid lower agent

*Physiotherapy:* Limb positioning, Passive stretching, Passive Range of Motion exercises followed by active assist, sit to stand, weight shifting in sitting and standing, assisted ambulation, bridging

**Between 2009-2018:**

Patient tried Acupuncture therapy and Acupressure therapy along with home-based based exercises

*Acupuncture Therapy:* Stimulation of defined anatomical location on skin. It is believed that stimulation with metallic needles release neuro-transmitters affecting structures of brain.

*Acupressure Therapy:* Use of finger pressure over the meridian points of the body, applying structured pressure or vibration to soft tissue structures, which might result in increased blood flow, release muscle spasm and release beta endorphins.

**Details at the time of Enrolment (2018):**

*Chronicity:* 9 years *Lesion volume in MRI*: 11.7 cm^3^

*Neck Control* – Good *Trunk Control* - Good

*Manual Muscle Test (MMT):* 3-  *Hypertonia at EDC muscle*: 2

*Gait*: Circumductory *DTR* 3+ for Brachioradialis

*Visual Analog Scale (VAS) for pain:* 3  *Mini Mental Examination Score:* 30

*Cerebellum:*

Finger to Nose test (FNT): Normal Heel to Shin test (HTS): Normal

*Sensation:*

Superficial (pain / touch / temperature / pressure) - Present

Deep (proprioception / kinesthesis / Vibration) - Present

Cortical (Tactile Localization / Stereognosis / Graphesthenia / Barognosis / 2-point discrimination / Texture recognition) - Present

Modified Ashworth Scale at wrist joint: 2

Brunnstrom Stages: 4

Modified Rankin Scale : 2

Barthel Index: 85

Passive ROM: 45^0^

Active ROM: 25^0^

*Upper Limb Fugl Mayer:*

Upper extremity: 28/36, Wrist: 2/10, Hand 7/14, Coordination Speed: 6/6, Sensation: 6/12, Passive Joint Motion: 24/24, Joint Pain: 24/24

*Lower-Limb Fugl Mayer:*

Lower Extremity: 23/28, Coordination/ speed: 6/6, Sensation: 12/12, Passive Joint Motion 20/20, Joint Pain: 20/20

**Details of physiotherapy suggested at the time of enrolment:**

- Tasks specific to upper extremity included:
- Passive stretching of long wrist flexors with the hold of 30 sec followed by relaxation
- Fist making in prone and thrust release-10 reps (3sets)
- Fist making in mid prone position -10 reps (3sets)
- Ball squeezing and release-10 reps
- Lock and key movements -10 reps
- Muscle facilitation techniques like stroking, brushing, icing - 3-5 strokes twice in a day
- Task-oriented training (TOT); reaching, grasping a tool, lifting a glass of water, pen holding, bottle opening.
- The task was made challenging by adding gradations like:
- Gross to fine movements
- Static to dynamic positioning
- Single to dual activity
- Non weighted to the incorporation of weights
- Indoor to the outdoor environment
- In case the patient experiences any kind of pain or fatigue in wrist/hand, proximal joints like shoulder/elbow; balance or coordination difficulties while doing a particular task, the task was fragmented in small segments for the ease of practice and was gradually built up from there for achieving the required functional goal.

**Other Details at the time of Post-therapy (other than in Manuscript):**

*Upper Limb Fugl Meyer:*

Upper extremity: 31/36, Wrist: 5/10, Hand 10/14, Coordination Speed: 6/6, Sensation: 6/12, Passive Joint Motion: 24/24, Joint Pain: 24/24

*Lower-Limb Fugl Meyer:*

Lower Extremity: 25/28, Coordination/ speed: 6/6, Sensation: 12/12, Passive Joint Motion 20/20, Joint Pain: 20/20

***
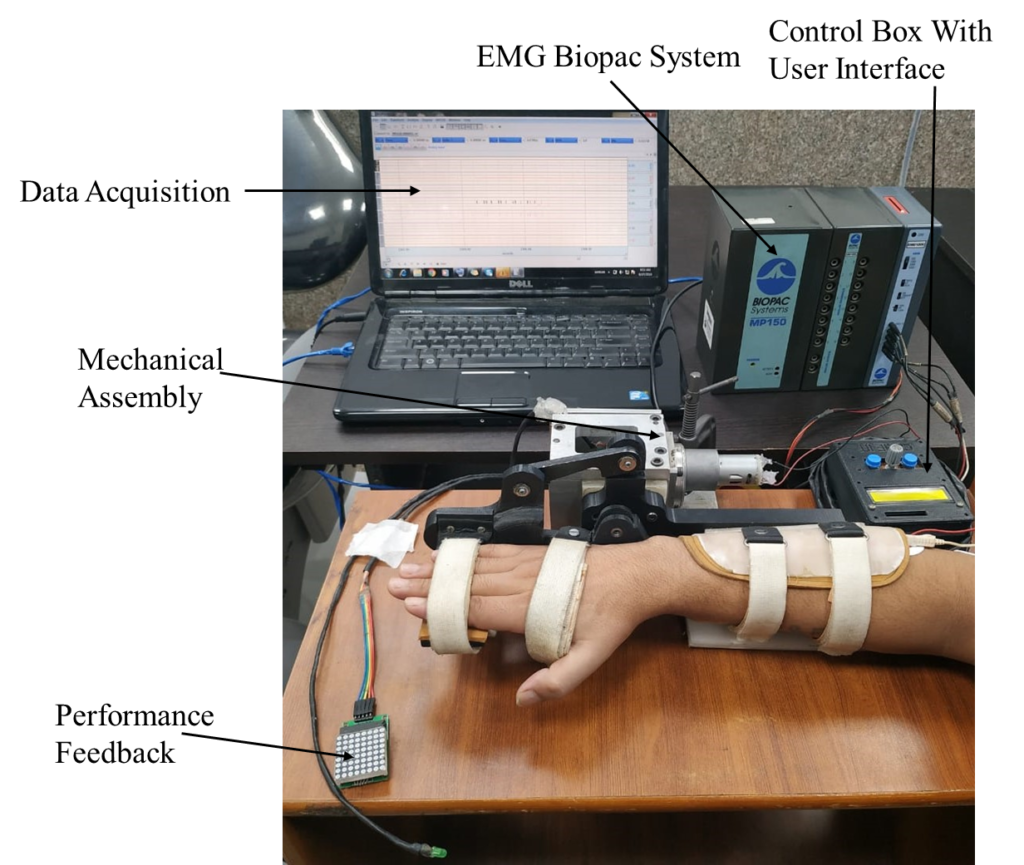
 Supplementary Figure 1: Robotic Exoskeleton with full set-up***

| ***Supplementary Table 1 Subjective Questionnaire Feedback form filled by the patient***  (4) | | |
| --- | --- | --- |
| **S.No.** | **Questionnaire** | **Answers** |
| 1 | Was the robotic hand comfortable to wear and take it off? Were you able to wear it individually using the other hand? | **Yes** |
| 2 | Was it user-friendly? Were you able to operate it individually, once told? | **Yes** |
| 3 | Was it simple and interactive? | **Yes** |
| 4 | Were you able to understand its procedure and synchronize with it? | **Yes** |
| 5 | Was it tedious to perform this specific activity for a longer duration? | **No** |
| 6 | Did you experience any tiredness or fatigue during the protocol duration? If yes, please specify the area of your hand (draw the hand area) where you felt any pain or fatigue. | **Yes, 0.5 hour** |
| 7 | Mention the duration after you felt fatigued or in pain. | **No pain** |
| 8 | With the arm positioned in a robotic hand, did you encounter any sort of positional stress in any joint or part of the forearm? | **No** |
| 9 | At any point in time did you encounter any fear of being hit by robotic hand? If yes describe it. | **No** |
| 10 | Do you feel comfortable and safe enough to use it on your own without being supervised by your therapist? If not, why? | **Yes** |
| 11 | Do you see any limitations? If yes, please comment. | **No** |
| 12 | Would you like to recommend it to other patients who have limitations in hand functionality? | **Yes** |

| ***Supplementary Table 2 System Usability Scale*** (4) | |
| --- | --- |
| **Questionnaire Items** | **Score** |
| 1. I think that I would like to use this system frequently. | **4** |
| 1. I found the system unnecessarily complex. | **1** |
| 1. I thought the system was easy to use. | **5** |
| 1. I think that I would need the support of a technical person to be able to use this system. | **1** |
| 1. I found the various functions in this system were well integrated. | **1** |
| 1. I thought there was too much inconsistency in this system. | **1** |
| 1. I would imagine that most people would learn to use this system very quickly. | **5** |
| 1. I found the system very cumbersome to use. | **1** |
| 1. I felt very confident using the system | **5** |
| 1. I needed to learn many things before I could get going with this system. | **1** |
| **SUS Score** | **87.5** |

***Subjective questions asked:***

**Question**: Whether you were aware of the robotic hand therapy after stroke in India or abroad?

**Answer**: “Yes, I had heard about robotic hand about four years back in Australia. There I visited also but the robotic hand did not fit my hand. So, I had to return without using it.”

**Question**: How was your experience with the robotic hand therapy you underwent here?

**Answer**: “The robotic hand movements gave me new hope for wrist movement which has become much softer now, and the chance of improvement has now really increased despite me being so chronically ill for the last nine years.”

**Question**: Can you think of it as a home-based rehabilitation device for patients with stroke or any other patients who have hand impairments?

**Answer**: “Yes, I think it’s a big hope for everybody who is suffering from stroke to do their own therapy. It is a very simple and user-friendly therapy. The patients at home can use it very well. It will give them a lot of confidence as you don’t have to depend on somebody else and can do your own exercise of the wrist and make it soft when it matters the most in the first few years after stroke.”

**Details of data acquisition through Transcranial Magnetic Stimulation (TMS):**

Mrs. X was compliable with Transcranial Magnetic Stimulation (TMS). She sat comfortably on the chair, kept forearm pronated, elbow-joint at 90–120° flexion, wrist-joint at a neutral position, and fingers at rest. The disposable gel-based wet Ag/AgCl surface electrodes were used in a bipolar configuration in which active electrodes were placed on the muscle belly of Extensor Digitorum Communis (EDC) with a center-to-center inter-electrode distance of 20 mm and ground electrode was placed on the lateral epicondyle. Muscle contraction causing extension of third digit of hand was observed for identification of muscle-belly and electrode placement. Electrodes were connected to the EMG amplifier connected with TMS (Magstim Rapid^2^, Magstim, UK). Motor Evoked Potential (MEP) was acquired in a quiet place, the patients were instructed to close the eyes and continue deep breaths, keep the hand in a fully relaxed condition for 120 s before starting the experiment. The experiment was done at the same time of the day to ensure same experimental conditions for all the subjects. Specific hotspot for the EDC muscle was determined. Single-pulse TMS stimuli at 100% motor threshold were applied with the procedure widely used (6), using a flat 70 mm figure-of-eight coil (type-D70 (AC), serial no. 0326, Magstim Rapid^2^, UK) placed tangentially with handle pointing towards back, 90° to central sulcus and 45° to midsagittal line for trans-synaptical activation of the cortico-spinal tract (42). TMS stimuli were delivered by moving the coil in millimeters in all directions until the hotspot, producing maximum MEP response, was localized. RMT was defined as the minimum intensity of TMS at the hotspot required to elicit an MEP in target resting contralateral-muscle in 5/10 trials, recorded in EMG, over the muscle cortical representation in the primary motor cortex. Once the hotspot was localized, RMT was measured by progressively increasing the maximum stimulator output (MSO) starting from stimulus intensity of 35% in steps of 2 to 5% until a reliable MEP (>50 μV peak-to-peak) appears (6). Then, MSO is lowered in steps of 1% until there are 5 consecutive responses out of 10 trials. Each pulse were given at an interval between each stimuli of > 5 sec (43). Five MEP signals out of 10 consecutive trials were averaged. Cortical-excitability measures, RMT and MEP on cortical representation area of EDC muscle between Cz and C3/C4 of contralateral primary motor cortex with reference to the Electroencephalogram (EEG) cap for both the hemispheres. Once the hotspot was identified, MEPs were obtained in single trial from both the hemispheres.

***
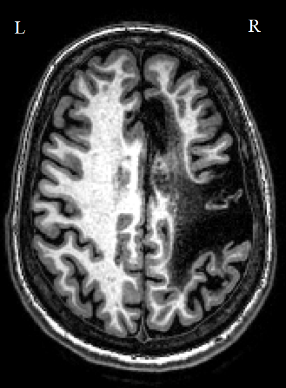
***

***Supplementary Figure 2: T1 weighted image in axial orientation of the patient showing ACA***
